# On a Case of Poisoning by Two Ounces of Arsenic; Recovery

**Published:** 1853-01

**Authors:** Thomas Bryant


### SELECTIONS.

From the London Lancet.

*On a Case of Poisoning by Two Ounces of Arsenic; Recovery.* By THOMAS BRYANT, M. R. C. S., &c.

W. C., aged thirty, single, a butcher, and a notorious drunkard, after having received some act of apparent unkindness from an aunt from whom he had "expectations," was induced to poison himself; and on the evening of July 11th, at half-past nine P. M., I was called to see him. On my arrival I found him quite drunk, could not gain any information from the friends present, and only elicited from him that he had taken about three grains of arsenic a quarter of an hour previously. As there were no symptoms of poisoning present, I returned home and gave him, immediately, an emetic of a scruple of ipecacuanha with one grain and a half of tartarized antimony. On my visit to him at half-past eleven P. M., I learnt, from a friend who was absent when I first called, that he had certainly taken *at least two tablespoonfuls of arsenic.* The emetic had acted immediately, in some measure had sobered him, and had brought up a quantity of dark-brown flaky fluid, which, on analysis, contained abundant arsenic. The man was very drowsy; skin moist; pupils natural; complained of pain on pressure over the pit of the stomach; tongue foul, but not injected; bowels purged once of a very fluid, fœtid stool; pulse 100, full and strong. After hearing the true history of the case, and as there were no symptoms of vomiting, I applied the stomach-pump, injecting barley-water (the only thing at hand) till it returned almost clear. The fluid withdrawn was more opaque, contained some brown curdy material and some arsenic, but not more than ten grains. Not feeling satisfied with this result, I ordered one scruple of the sulphate of zinc every two hours until I saw him at half-past eight A. M. the following morning. I then found that he had taken four doses of the zinc, had vomited considerably after each dose, but not continually; the fluid rejected was clear, with brown curdy flakes suspended in it, (containing arsenic,) and on pouring off the supernatant fluid, the arsenic collected was about sufficient to cover a shilling. The man was bathed in sweat, experienced slight pain in the abdomen, increased on pressure, but at times very severe; there was but little dryness of the throat; tongue foul and slightly injected; bowels purged three times; the stools loose and of a dark color; pulse 100, full, but weak; and the patient expressed himself as feeling "tolerably well." The hydrated oxide of iron was given in doses of two ounces every two hours, and a dose of castor-oil exhibited. Nine P. M.: Nausea, but no vomiting since eleven A. M.; still slight abdominal pain; tongue foul, but very slightly injected; bowels have been open three times with pain; motions very offensive, of a dark color, and one looked bloody; pulse 100, full, and of more power.

July 13th.—Twelve at noon: Passed a good night; has still pain over the abdomen, increased on pressure; tongue the same; bowels have acted twice, but the stools were thrown away; skin moist; no perceptible dryness or redness of the throat; pulse 96, full, and of good power; indeed, the man says he is "nearly well." Continue with medicine.

14th.—Altogether improved; pain in abdomen less; skin moist; tongue cleaner and less injected; no nausea; bowels open twice, and motions loose, very offensive and dark colored; pulse the same. Ordered, spirit of nitrous ether, two drachms, tincture of henbane, one drachm and a half, water, six ounces; take one-fourth three times a day.

15th.—Still improving; no tenderness of the abdomen; tongue cleaner; bowels have not been opened; pulse 96, and natural. Castor-oil, one ounce at bed-time; repeat mixture.

16th.—Bowels opened four times, and motions contained scybalæ and blood; there is no pain in the abdomen, not even on severe pressure; tongue foul, but not injected; skin cool; pulse natural. Repeat oil.

17th.—Bowels opened twice, stools depositing a white powder, and contained blood; otherwise much improved.

18th.—Stools natural, but loose.

21st.—Has continued daily to improve; motions healthy and solid; and indeed may be considered well.

*Remarks.*—The questions which naturally arise in the mind of any one on the perusal of the above case would be, first,—Did the patient take *arsenic?* and secondly, What *quantity* did he really take? Now, in answer to these natural queries, I must add, that a friend of the man had a few days previously given him a packet containing about *four ounces* of arsenic, for the purpose of destroying vermin; that this packet had been placed upon a certain shelf in the stable, and that the friend had seen it there unopened on the morning of the day of the attempted suicide. On the discovery of the attempt, this same paper containing the arsenic was found at the feet of the patient, he having dropped it on hearing some one enter his room, with only about *one* tablespoonful in it. This was immediately taken by the party who discovered it to a neighboring chemist, who, unfortunately, after mixing it in water, and not knowing what it was, or its history, threw it away on some stones before his shop.

Now, here seems a tolerably clear account that the packet contained originally about *four ounces;* none had been used, as the patient afterwards informed me; that little had fallen in the room, as none had been noticed; and the small remainder leaves a deficit of between two or three ounces; and added to this, there is the firm assertion of the friend who had been with him, and gave him originally the poison, that the quantity at least taken was two tablespoonfuls; and the man himself says that he took all that was in the packet originally, with the exception of what was found afterwards. The quantity which was left was specified to me by the chemist and the man who took it to him, on several different occasions, as certainly not more than one tablespoonful.

Not feeling satisfied with this account, I thought some mistake must have been made with respect to the strength of the powder taken. I therefore had the powder which the chemist had thrown away upon the flag-stones before his shop collected, and through the kindness of Dr. Odling, of Guy's Hospital, who analysed it for me, learnt that it contained ninety-five per cent. of arsenious acid, the remainder being sand, which most probably was added by the scraping of the stones during the collection of the powder. Being then obliged to recognise the fact that at least two ounces of arsenious acid had been swallowed, the grand difficulty remains, to account for the mildness of the symptoms which a large dose of such an irritant poison would naturally be expected to occasion. Such a result I find quite unable to accomplish, and only suggest the question, whether the man, from his habit of habitual drunkenness, could so have thickened, and therefore rendered less sensitive, the lining membrane of his stomach, and thus have given more time for the means which were employed to rid that organ of its poisonous contents? Still this will not account for the absence of some symptoms which would have been expected, nor for the mildness of all those present; and I can only assign this case, as we are obliged to many others of different characters, to that class which we call anomalous.

